# Identification and real-time expression analysis of selected *Toxoplasma gondii in-vivo* induced antigens recognized by IgG and IgM in sera of acute toxoplasmosis patients

**DOI:** 10.1186/1471-2334-13-287

**Published:** 2013-06-24

**Authors:** Atefeh Amerizadeh, Boon Yin Khoo, Ai Ying Teh, Majid Golkar, Izzati Zahidah Abdul Karim, Sabariah Osman, Muhammad Hafiznur Yunus, Rahmah Noordin

**Affiliations:** 1 Institute for Research in Molecular Medicine (INFORMM), Universiti Sains Malaysia (USM), Penang 11800, Malaysia; 2 Parasitology Department, Molecular Parasitology Laboratory, Pasteur Institute of Iran, Tehran, Iran

**Keywords:** *Toxoplasma gondii*, *In-vivo* induced antigen technology (IVIAT), cDNA library immunoscreening, Acute toxoplasmosis sera, mRNA expression analysis, Real-time polymerase chain reaction (PCR)

## Abstract

**Background:**

*Toxoplasma gondii* is an obligate intracellular zoonotic parasite of the phylum Apicomplexa which infects a wide range of warm-blooded animals, including humans. In this study *in-vivo* induced antigens of this parasite was investigated using *in-vivo* induced antigen technology (IVIAT) and pooled sera from patients with serological evidence of acute infection.

**Methods:**

The pooled sera was first pre-absorbed against three different preparations of antigens from *in-vitro*-grown cells of each *T. gondii* and *E. coli* XL1-Blue MRF’, subsequently it was used to screen *T. gondii* cDNA phage expression library. Positive clones from each group were subjected to quantitative real-time PCR expression analysis on mRNA of *in-vivo* and *in-vitro* grown parasites.

**Results:**

A total of 29 reactive clones from each IgM and IgG immunoscreenings were found to have high homology to *T. gondii* genes. Quantitative real-time PCR expression analysis showed that 20 IgM-detected genes and 11 IgG-detected genes were up-regulated *in-vivo* relative to their expression levels *in-vitro.* These included genes encoding micronemes, sterol-regulatory element binding protein site, SRS34A, MIC2-associated protein M2AP, nucleoredoxin, protein phosphatase 2C and several hypothetical proteins. A hypothetical protein (GenBank accession no. 7899266) detected by IgG had the highest *in-vivo* over *in-vitro* fold change of 499.86; while another up-regulated hypothetical protein (GenBank accession no. 7898829) recognized by IgM showed high sensitivity (90%) and moderate specificity (70%) in detecting *T. gondii* antibodies when tested with 20 individual serum samples.

**Conclusion:**

The highly up-regulated genes and the corresponding proteins, in particular the hypothetical proteins, may be useful in further studies on understanding the disease pathogenesis and as potential vaccine candidates.

## Background

Toxoplasmosis is caused by the zoonotic and ubiquitous *Toxoplasma gondii*, with up to 30% of the world human population affected by this parasite. Infection usually occurs by ingestion of *T. gondii* oocysts from infected cats or consumption of undercooked meat containing the parasite cysts. Environmental, cultural factors and eating habits are thought to be contributing factors in the transmission of this infection [1-4]. The foetus of an infected mother can acquire this infection by vertical transmission through the placenta during early pregnancy. In congenital toxoplasmosis most of the mothers and newborns are asymptomatic but severe sequelae may develop later in the infant life such as inflammatory lesions, mental retardation, seizures and choriorentitis with or without hydrocephaly. Prompt treatment of the affected child would be possible if diagnosed early [5-8]. Diagnosis of toxoplasmosis is usually performed by detection of IgG and IgM against *T. gondii*. In addition, IgG avidity test is an important additional test that is routinely performed; with low IgG avidity suggesting acute infection while high IgG avidity confirms chronic infection [9].

*In-vivo* induced antigen technology (IVIAT) is a new and promising method introduced by Martin Handfield *et al.* in 2000 which can be used to determine *in-vivo* induced antigens that are directly related to the human infection and thus reduce false-positive results caused by the differences between proteins expressed during *in-vitro* culture and actual human infection [10]. IVIAT uses sera from patients or animals infected with the pathogen of interest and therefore obviate the need for animal models. Up-regulation of identified genes by IVIAT can be assessed by techniques such as quantitative real time PCR (RT-PCR) or microarray [10-12]. In a previous study, our group has used IVIAT to identify *in-vivo* induced genes of *T. gondii* which expresses proteins reactive with Toxoplasma specific IgG antibodies in chronically-infected individuals [13].

In the present study we applied IVIAT to identify *in-vivo* induced antigens of *T. gondii* using sera of acutely-infected patients with low anti-Toxoplasma IgG avidity and high IgM positivity. These antigens may be potentially useful as diagnostic markers, vaccine candidates or in increasing our understanding of the disease pathogenesis.

## Methods

### Parasite strain and growth conditions

*In-vitro* culture of *T. gondii* RH strain in Vero cells was performed under conditions previously optimized in our laboratory [14]. Vero cells were washed four times at 85% confluence with phosphate buffered saline (PBS), followed by addition of DMEM medium (Gibco BRL, USA) containing 100 μg/ml streptomycin and 100 IU/ml penicillin (Gibco BRL, USA) with 10% (v/v) fetal bovine serum (Invitrogen, USA). Subsequently the cells were seeded with 1x10^7^*T. gondii* tachyzoites harvested from infected mice. After 3–4 days the maximum release of tachyzoites was observed and the culture containing parasites was centrifuged and the pelleted tachyzoites was kept at −80°C.

To produce *in-vivo* grown tachyzoites, Swiss albino mice were intraperitoneally infected with 1 × 10^3^ tachyzoites of *T. gondii* RH strain. After three to four days post-infection, the peritoneal cavity fluid was aseptically harvested with 5 ml of RPMI-1640 medium containing penicillin streptomycin (RPMI-PS), pH 7.2 (Gibco®, Life Technologies, USA). The supernatant containing tachyzoites was collected, centrifuged, washed with PBS and pelleted tachyzoites immediately kept at −80°C for RNA extraction. Approval from the USM Animal Research Ethics Committee was obtained prior to performing the animal infection.

### Serum samples

Commercial IgM and IgG ELISA kits (Euroimmun, Germany) were used to determine the Toxoplasma serology status of the serum samples in this study. Twelve sera samples were obtained from patients with clinical evidence of toxoplasmosis. Toxoplasma serology performed on the serum samples showed high IgM antibody levels and low IgG avidity indices. These sera were collected by one of the co-authors from Molecular Parasitology Laboratory, Parasitology Department, Pasteur Institute of Iran. Ethical clearance from the institution and informed consents from patients were obtained. Prior to performing IVIAT, equal volume of each serum samples was pooled. Out of a total of 12 serum samples, 10 were individually used to determine sensitivity of selected clones. In addition, another 10 serum samples from healthy individuals which were negative for both anti-Toxoplasma IgM and IgG antibody were individually used to determine specificity of selected clones.

### Sera pre-adsorption

Sera adsorption was performed according to previously reported protocol with some slight modifications in order to increase efficiency of the process so as to ‘completely’ remove *in-vitro* antigens [13,15]. One modification was to increase the incubation of pooled sera with each of cell pellet, heat denatured and non-heat-denatured cell lysates of *T. gondii* &*E. coli* XL1-Blue MRF’ from 1 h to overnight at 4°C on a shaker. Each step was repeated twice and thimerosol (1%) was added in order to prevent contamination. Serum samples used for sensitivity and specificity determination (section 2.8) were preadsorbed with pellet, heat denatured and non-heat-denatured cell lysates of only *E. coli* XL1-Blue MRF’. The efficiency of sera adsorption at each step was checked using indirect enzyme-linked immunosorbent assay (ELISA) according to the previously optimized protocol [13]. The final adsorbed sera sample was centrifuged and kept at −80°C.

### T. gondii expression library

Custom-made *T. gondii* cDNA expression library was constructed in Lambda ZAP® II system (Strategene, USA) using mRNA from *in-vitro* grown *T. gondii* pellet. The previously optimized dilution of cDNA library immunoscreening was 10^3^ for primary screening which produces around 500–1000 plaques per plate. For secondary and tertiary screening 10^4^ and 10^5^ dilutions of cDNA library were used respectively.

### cDNA library immunoscreening

For *T. gondii* cDNA library screening a modified version of a previously reported method was used [16]. For primary screening, the 10^3^ dilution of cDNA library was mixed with 600 μl of *E. coli* XL1-Blue MRF’ cells at OD_600nm_ 0.5, then plated on LB agar plates 3–4 h in a 42°C incubator until the plaques became visible. On the next day, the agar plates were overlaid with nitrocellulose filter disc pre-saturated with 10 mM isopropyl-β-D-thiogalactopyranoside (IPTG) and incubated overnight at 28°C. The filters were then removed from the plates, washed (colony side up) with PBS-Tween 20, blocked with 1:5 dilution of Super Block solution (Thermo Scientific, USA) and incubated overnight with adsorbed pooled serum at dilution 1:100. Subsequently after three step of washing with PBS-Tween 20 the filters were incubated with peroxidase-anti-human IgG (Invitrogen, USA) at 1:3000 dilution or peroxidase-anti-human IgM (Invitrogen, USA) at 1:2000 dilution for 1 h, washed and then developed using chemiluminescence substrate (Roche Diagnostics, Germany). The phage plaques on the agar plates which corresponded to positive plaque images on the X-ray film were cored out and subjected to secondary and tertiary screenings using the same serum. For secondary and tertiary screenings, higher dilutions were used to produce much lower density plaques (<50 per plate) with many isolated clones. Final positive clones were isolated and placed in 500 μl SM buffer containing 3% chloroform overnight at 4°C. Part of them were used directly for *in-vivo* excision, while the rest were centrifuged and the supernatant placed in 500 μl SM buffer with 7% DMSO for long-term storage at −80°C.

### *In-vivo* excision and plasmid purification

*In-vivo* excision was performed to convert the recombinant phages to recombinant plasmids. The selected phage clone (in 250 μl SM buffer) and 1 μl ExAssist helper phage (>106 pfu/ml) (Stratagene, USA) was added to 200 μl of *E. coli* XL1-Blue MRF’ cells at OD_450nm_ 1.0. The culture was incubated at 37°C for 15 min followed by addition of 3 ml of LB broth and incubation in 65°C for 20 min. After centrifugation 10 μl of supernatant (excised phagemids) was mixed with 200 μl of SOLR cells (Stratagene, USA) at OD 1.0 (405 nm) and plated overnight at 37°C on LB ampicillin agar plate. Plasmids from each sample was purified using commercial plasmid preparation kit (Promega, USA) and sent for sequencing using M13 forward and reverse primers. The sequences identified by IVIAT in current study after sequencing were then analyzed using GenBank and ToxoDB (http://toxodb.org/toxo) databases. ToxoDB, a functional genomic database for *T. gondii* which incorporates sequence and annotation data, is integrated with other genomic-scale data, including community annotation; expressed sequence tags (ESTs) and gene expression data [17].

### Quantitative real-time PCR analysis

The forward and reverse primers for each of the sequences were designed using Primer Express 2.0 software (Applied Biosystems, USA). Table 1 shows the primers designed based on sequences of IgM-detected genes; while Table 2 shows the primers designed based on sequences of IgG-detected genes. Pooled *in-vitro*-grown tachyzoites from 20 culture flasks (75 cm^2^) (Nunclon, Roskilde, Denmark) and pooled *in-vivo*-grown tachyzoites from 35 mice were prepared as described above. Total RNA was extracted and purified using RNAeasy mini kit (Qiagen, Germany). The DNase-treated RNA samples were converted to cDNA using High Capacity cDNA Reverse Transcription Kits (Applied Biosystems, USA). Real-time PCR was performed using Quanti FastTect SYBR Green PCR Kit (Qiagen, Germany) in a Rotor Gene 6000 Multiplex System (Corbett Research, Australia). Each of the reaction mix comprised 5 μl of cDNA (200 to 400 ng), 200 pmol of each primer (forward and reverse) and 12.5 μl of 2× SYBR Green mix and sufficient amount of dH_2_O to bring the volume to 25 μl. The parameters for thermocycler were as follow: 95°C for 5 min short hot-start, followed by 40 cycles of 95°C for 10 sec for denaturing and subsequently combine annealing/extension at 60°C for 30 sec. At the final step of real-time PCR, melting curves with 1°C temperature increments from 72°C to 95°C were incorporated. Data analysis was performed using Rotor Gene 6000 Series Software 1.7. First the expression level was normalized to the reference β-actin gene as housekeeping gene. Next the fold change of *in-vivo* expression of the gene relative to its *in-vitro* expression level was calculated using 2^-^ΔΔ^Ct^ method [18].

**Table 1:** **Primers used in real-time PCR analysis of *T.gondii *IVIAT identified genes by IgM**

**GenBank ID/accession no.**	**Primer**	
	**Orientation sequence**	
^**^β*-Actin*	*F	5′-TCACACTGTGCCCATCTACGA-3′
	*R	5′-GTGGTGAAGCCGTATCCTCTCT-3′
**7895406**	F	5′-ACCGACTCGAAGCACAACAGT-3′
	R	5′-CATCGTAGGTGACGAACAATGC-3′
**7898829**	F	5′- CAATGTTTCGGGCAGCTCAT -3′
	R	5′- GCTGTACTGAAACCGTCGATGTAG -3′
**7894091**	F	5′- CCACTCAAGCAGTTCCTGAAGA -3′
	R	5′- GATCTCCGCGAAATCTGTCTCT -3′
**7900912**	F	5′- CGCATTTACAAGCGCCTGAT -3′
	R	5′- TTCCACGCCTCGTTTACATG -3′
**7899945**	F	5′- CTTCTCGACGACATGCATCTG -3′
	R	5′- TGGACGCACTGGATGTTCTG -3′
**7893976**	F	5′- AGCGCAGCAAGAACCAAGA -3′
	R	5′- TTCCTCAAGAAGGCCATGGA -3′
**7893824**	F	5′- CTCACGTACGAGGCTAGATCCA -3′
	R	5′- GTCCGCCAGGGCATTCTATT -3′
**7899621**	F	5′- AGGCACAGGCTCCACCATAC -3′
	R	5′- AGCTCCAGGACGCGTAAGAG -3′
**7901413**	F	5′- TCTGAAGCTGCAAGGTTCCA -3′
	R	5′- TTGCAGAGTAGGCTCGTCAAAC -3′
**7894150**	F	5′- TCCTCGAACGGCAGAGAAA -3′
	R	5′- CCCCTCAGGTGTGAAGACATCT -3′
**7895338**	F	5′- AGAGCCGGTAGCACTGTGTTC -3′
	R	5′- CGGTAACCCGGTGGACTTCT -3′
**7898197**	F	5′- GGATTTCGGCACGCTCAAT -3′
	R	5′- CAACTGTGGCTCCCGACTACTT -3′
**7895974**	F	5′-CCAAATCGGTTCGCTCATGT-3′
	R	5′-GCAAAGGCCGGAGAATTCAT-3′
**7900011**	F	5′-CACGCTAACTGTTTCAGGTACGA-3′
	R	5′-TACAGCAGCGTGTTCATTTGC-3′
**7895000**	F	5′-TCCAACATCCCGACCTGATC-3′
	R	5′-GCTTCGACCTTCGCATTCTTC-3′
**L06091.1**	F	5′-CAGACTCCTTGCTCTCTTCTTGAA-3′
	R	5′-GTTGTGTCACTACTGCGATTGTTG-3′
**7896316**	F	5′-AAGGTGCTTCATCCGCCATA-3′
	R	5′-GGTCTTGCATATCTCGCCATTC-3′
**7894239**	F	5′-GAACTGTTTCAATGCGTCGATT-3′
	R	5′-GTGATCCAATCGCACACTCTTTT-3′
**7896580**	F	5′-CCTTCTGGTTAGCCACATCTGAT-3′
	R	5′-GGCGACGAAGATGATTTTGAC-3′
**7899034**	F	5′-CCTGGAACGTCCTTTCTGGAT -3′
	R	5′-ATCTAACCGTCGCGCTGGTA -3′
**7899034**	F	5′-ATGTTCCCGATCCAGAGCAA-3′
	R	5′-GGTGTGGCTGTCTCCCTATTTT-3′
**AY707938.1**	F	5′-GAACGCGAACAATGAGTTTG-3′
	R	5′-CCGCTGGTCGAAATAATGATG-3′
**7894324**	F	5′-GTTAATCAGGAGCAGACCCGATA-3′
	R	5′-TAAGCTACCCCTGAGCAAGTTGT-3′
**AF364813.1**	F	5′-GGTGGAAAGTGAATACCGTGGAT-3′
	R	5′-CAGTGAGTCCGAATGCCAAAT-3′
**7900910**	F	5′-GCCTGCAACATGTCCAACTTC-3′
	R	5′-TCAGCGGCTAAGCGAAAAAG-3′
**7896316**	F	5′-CATACGGATAGGCGGTCATGTA-3′
	R	5′-GTATACGAACGCCCTGATGTTG-3′
**7897539**	F	5′-ATGCAAGACCCAAGGCAAAC-3′
	R	5′-TGTACGGAACGCTTCTTCGA-3′
**7895127**	F	5′-GCGAAAGTCAAACGGAATGG-3′
	R	5′-TTCTGCGCGGAGTCGTTATT-3′
**7896796**	F	5′-GCTTTTACCTGCTCGGGAAGT-3′
	R	5′-AACGCATCTCACACGGATAGC-3′

*F forward, *R* reverse.

**β*-Actin* was used as the housekeeping gene.

**Table 2:** **Primers used in real-time PCR analysis of *T.gondii *IVIAT identified genes by IgG**

**GenBank ID/accession no.**	**Primer**	
	**Orientation sequence**	
^**^β*-Actin*	*F	5′-TCACACTGTGCCCATCTACGA-3′
	*R	5′-GTGGTGAAGCCGTATCCTCTCT-3′
**7901108**	F	5′-AAAGATGCTCCACGCCAACT-3′
	R	5′-GTTTGTCCACGTTGCTTCCA-3′
**7901478**	F	5′-TGAGCCAGAAACCTGACAAACA-3′
	R	5′-ATCGCCTTTTCCGCTGATTT-3′
**7894322**	F	5′-TCCTAAAGCTCGGCGGAATT-3′
	R	5′-CGGTTTGGGCTTTCCAGTTT-3′
**7894761**	F	5′-AGGCGTTTCAACCGACTCTGT-3′
	R	5′-ACCTGTGGCACAACAAGACATC-3′
**7897485**	F	5′-CAAGCGTGAGTTCGAGCAATAC-3′
	R	5′-GGCTTCCCATTTCGGTCATA-3′
**7899395**	F	5′-TCGTGGTGGACAAGGTATAGGAT-3′
	R	5′- CCATAGTTGTCGGAAGAGTCGTT-3′
**7895435**	F	5′- GGGAAAGCATCTGGCTTCAA-3′
	R	5′- GAGGAACGGAAAGTAGCGGAAT-3′
**7895096**	F	5′-CTCGAAACGATCGCTGCTTT-3′
	R	5′-ACTCCCGCAGAGTTCAAGACTT-3′
**7895077**	F	5′-CCTTCCACAAGTTGGCACAGA-3′
	R	5′-CAGTAGCTCCTCAAGCAGTGACA-3′
**7897605**	F	5′-GATGATCGAAGCTCGCAAGTG-3′
	R	5′-TAGACAAGGTGCCGCATCAAC-3′
**7899217**	F	5′-GTTCCTCGAGCTTTCCAACCT-3′
	R	5′-AGAGCTGCATCTGGAGCAGAA-3′
**AM055942.3**	F	5′-GAGGCACCGCTTCTTCTATACTG-3′
	R	5′-TGGGATCGTGTAAGCCATAGC-3′
**7894058**	F	5′-TTTCCCTGCGGAGCAGTCT-3′
	R	5′-CGCCTGCAACCTGAGATGA-3′
**7893896**	F	5′-GCGAAAGGTCTGGAACATTTG-3′
	R	5′-GGCAGGTAGGAAAGGAAATCGT-3′
**7899266**	F	5′-GGCACTTGTCAATGCAGAACA-3′
	R	5′-CTAATTGACCGGTGCACGAAA-3′
**7893521**	F	5′-GGAGGTCATCCTGTGGACTCA-3′
	R	5′-GGCTCACGACTTTCACCTTCA-3′
**7893531**	F	5′-CGATACATGCGGCAGAGTCA-3′
	R	5′-TGCTGGCTCGTCACATTCAG-3′
**7896164**	F	5′-ATCTCGGGAGTCTTCAGCAGACT-3′
	R	5′-GACGGCTGTTTCTCGACTTTTC-3′
**7896097**	F	5′-TGTTGCTACCATGGGCCATT-3′
	R	5′-TGTATCTGTGCCGGTCTTCGT-3′
**7901507**	F	5′-CGTATTCAAGCGCTGTTCGA -3′
	R	5′-GAGACGATTGGCAGCGAATT -3′
**7894761**	F	5′-AACCGACTCTGTCGCATCTGT-3′
	R	5′-CAAACCTGTGGCACAACAAGA-3′
**7900407**	F	5′-GCCTTCCTCGTAACCCAACTT-3′
	R	5′-AAGCTTGCCAGAACGACGAA-3′
**7897848**	F	5′-CTGATTCGGGCACTGATTGA-3′
	R	5′-GACGCGAAAAAGAGGGCATA-3′
**7900113**	F	5′-CGAGTTCTTTCGCGCTTTCT-3′
	R	5′-ATGGTTGGCAGACGGTCAAG-3′
**7899268**	F	5′-TCAGCTCGGAAGCACAGAATC-3′
	R	5′-ACGTGTCAGGCGGTGAATACT-3′
**7893794**	F	5′-TCCCGAAGGGAAAAAGTAGTCATA-3′
	R	5′-TTTGTCTTGGACTTCCGTATACCA-3′
**7893464**	F	5′-CGAACCCTGCCAATTCTCAT-3′
	R	5′-TCCATCCCCTCAGCAAAGTT-3′
**7899477**	F	5′-CTGTGTAAGGCGAATTTCTTTCG-3′
	R	5′-AGAAGAGAATCTGGCACGATGTG-3′
**AY424892.1**	F	5′-CAGGAAGCCATGCACACTGT-3′
	R	5′-GGCGACTCGATCCATTCCTA-3′

^*^F forward, *R* reverse.

^**^β*-Actin* was used as the housekeeping gene.

### Sensitivity and specificity determination

To determine the sensitivity and specificity of selected clones, one clone was plated per petri dish and immunoscreening was performed as above with the exception that individual serum samples from acutely infected patients were used instead of pooled sera. Each nitrocellulose membrane was divided into six sections i.e. for six different serum samples. Positive and negative controls were included in each experiment. Sensitivity refers to the number of serum samples from acute-infected individuals (n = 10) which were reactive with the protein expressed by a particular clone. Thus if 8 serum samples are reactive out of 10, the sensitivity of the clone in the detection of *anti-Toxoplasma* antibodies in that group of serum samples is 8/10 or 80%. Specificity refers to the number of serum samples from healthy individuals (n = 10) which were not reactive with the protein expressed by a particular clone. Thus if 9 serum samples are not reactive out of the 10, the specificity of the clone in the detection of *anti-Toxoplasma* antibodies is 9/10 or 90%.

## Results

### Adsorption of pooled sera from patients with acute toxoplasmosis

**Figure 1:**
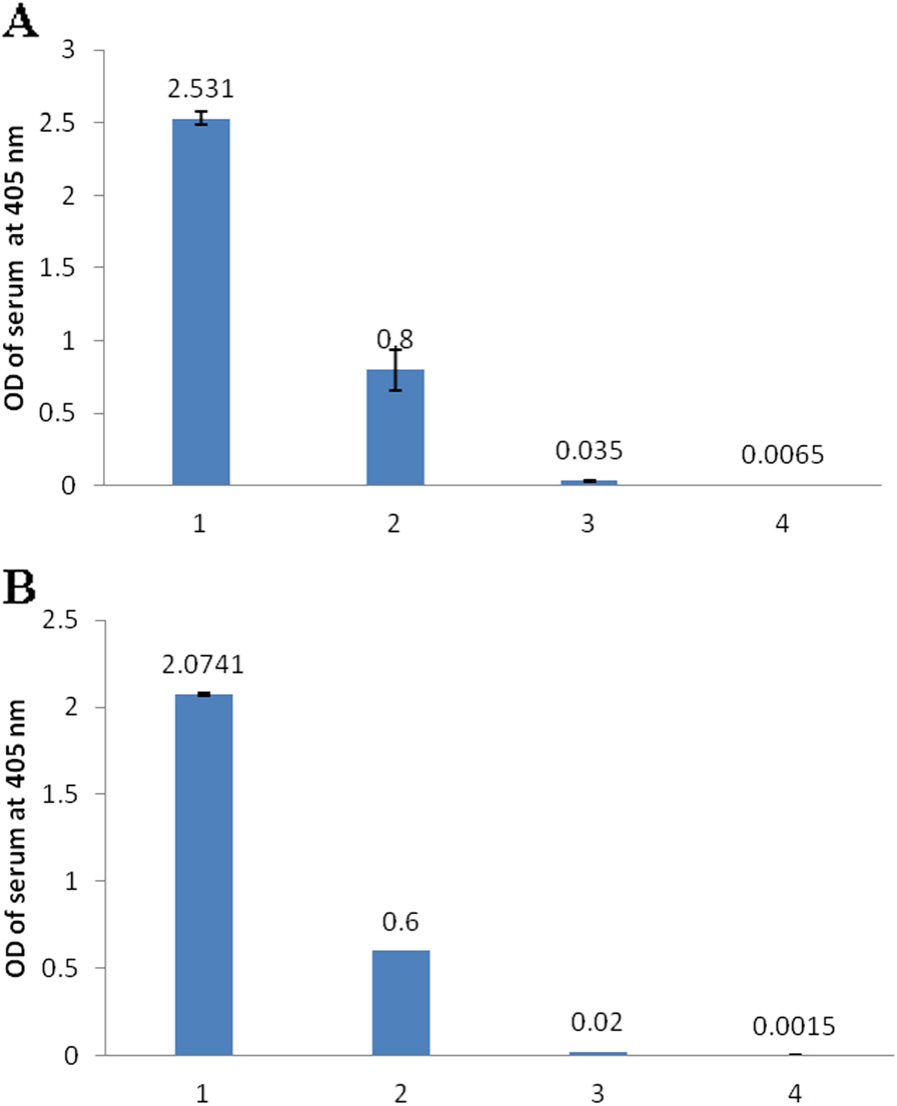
**Optical densities (OD) of pooled serum adsorped by *E. coli *and *T. gondii*. (A)** serum adsorped by three different forms of *E. coli* XL1-Blue MRF’, and **(B)** serum adsorped by three different forms of *T. gondii*. **1**-pooled non-preabsorped serum (control); **2**-serum adsorped by whole cell; **3**-serum adsorped by non-heat denatured cell lysate; and **4**-serum adsorped by heat denatured cell lysate.

Figures 1A and 1B show that the pooled sera were exhaustively pre-adsorbed against *T. gondii* and *E. coli* XL1-Blue MRF’ in-vitro antigens. Most of the reductions in OD values were observed after pre-adsorption with the pellet of the organisms. After the last step of serum pre-adsorption, the OD values of 0.001-0.009 were observed for serum diluted 1:100, which was the dilution used in the immunoscreening step.

### cDNA library tittering, screening and clone isolation

Approximately 10 000 and 14 000 clones from *T.gondii* recombinant phage cDNA library were screened with HRP-conjugated anti-human IgM and IgG respectively. A total of 35 clones were randomly selected from about 200 positive clones after the three stage immunoscreenings (primary, secondary and tertiary) with anti-human IgM-HRP. After sequencing, 29 clones showed high homology (80-100 %) to *T. gondii* genes (Table 3). Meanwhile a total of 38 clones were randomly selected from about 300 positive clones after the three stage immunoscreenings with anti-human IgG-HRP. After sequencing, 29 clones showed high homology (80-100%) to *T. gondii* genes (Table 4).

**Table 3:** ***Toxoplasma gondii *IVIAT- identified genes by immunoscreening of cDNA library using anti-human IgM-HRP**

**GenBank ID/accession no.**		**Gene ID in Toxo DB**	**Gene name**	**Description**	**Homology percentage**
**1**	7895406	TGME49_026460	hypothetical protein	*Toxoplasma gondiiME49* hypothetical protein	99%
**2**	7898829	TGME49_057020	hypothetical protein	*Toxoplasma gondiiME49* hypothetical protein	86%
**3**	7894091	TGME49_030160	hypothetical protein	*Toxoplasma gondiiME49* hypothetical protein	97%
**4**	7900912	TGME49_066920	phosphodiesterase	*Toxoplasma gondii ME49* phosphodiesterase, putative, mRNA	97%
**5**	7899945	TGME49_023050	40s ribosomal protein S20	*Toxoplasma gondii ME49* 40s ribosomal protein S20, putative	99%
**6**	7893976	TGME49_046620	G10 protein	*Toxoplasma gondiiME49* G10 protein, putative	99%
**7**	7893824	TGME49_066140	sterol-regulatory element binding protein site	*Toxoplasma gondii ME49* sterol-regulatory element binding protein site 2 protease, putative, mRNA	83%
**8**	7899621	TGME49_060190	microneme protein	*Toxoplasma gondiiME49*	95%
				Toxoplasma gondii strain RH microneme protein 13 (MIC13) gene, complete cds	
**9**	7901413	TGME49_004530	microneme protein	*Toxoplasma gondii ME49* microneme protein TgMIC11 precursor (MIC11) mRNA, complete cds	80%
**10**	7894150	TGME49_001880	hypothetical protein	*Toxoplasma gondiiME49* hypothetical protein	81%
**11**	7895338	TGME49_071740	hypothetical protein	*Toxoplasma gondiiME49* hypothetical protein	80%
**12**	7898197	TGME49_086550	hypothetical protein	*Toxoplasma gondiiME49* hypothetical protein	81%
**13**	7895974	TGME49_048600	aspartate aminotransferase	*Toxoplasma gondii ME49* aspartate aminotransferase, putative, mRNA	91%
**14**	7900011	TGME49_093510	poly(ADP)-ribose polymerase domain-containing	*Toxoplasma gondiiME49* poly(ADP)-ribose polymerase domain-containing	99%
**15**	L06091.1	TOXGRA5A	dense granule precursor (GRA5) gene	*Toxoplasma gondii strain RH* clone pcGRA5-IR dense granule antigen	100%
**16**	AM055943.1 7896316	TGME49_008440	cytoplasmic protein Cyt16	*Toxoplasma gondii RH* genomic DNA chromosome Ib, cytoplasmic protein Cyt16 (cyt16) gene, complete cds	93%
**17**	7894239	TGME49_054870	hypothetical protein	*Toxoplasma gondiiME49* hypothetical protein	89%
**18**	7896580	TGME49_008830	hypothetical protein	*Toxoplasma gondiiME49* hypothetical protein	91%
**19**	7899034	TGME49_057360	Hypothetical protein	*Toxoplasma gondiiME49* hypothetical protein	99%
**20**	7899034	TGME49_057360	hypothetical protein	*Toxoplasma gondiiME49* hypothetical protein	99%
**21**	AY707938.1	TGME49_071050	SRS34A (= SAG2A, P22)	*Toxoplasma gondii ME49* surface antigen P22 3′ flanking sequence	100%
**22**	7894324	TGME49_030180	hypothetical protein	*Toxoplasma gondii ME49* hypothetical protein	98%
**23**	AF364813.1	TGME49_014940	MIC2-associated protein M2AP	*Toxoplasma gondii ME49* MIC2-associated protein precursor, gene, complete cds	99%
**24**	7900910	TGME49_066900	cyclin, N-terminal domain-containing protein	*Toxoplasma gondiiME49* cyclin, N-terminal domain-containing protein, mRNA	100%
**25**	7895521	TGME49_058470	Hypothetical protein	*Toxoplasma gondiiME49* hypothetical protein	99%
**26**	AM055943.1 7896316	TGME49_008440	hypothetical protein	*Toxoplasma gondii RH* genomic DNA chromosome Ib	98%
**27**	7897539	TGME49_116540	Hypothetical protein	*Toxoplasma gondiiME49* hypothetical protein	97%
**28**	7895127	TGME49_027280	dense granule protein 3	*Toxoplasma gondii ME49* dense granule protein 3, mRNA	90%
**29**	AM055943.1 7896796	TGME49_121620	dynamic-like protein	*Toxoplasma gondii RH* genomic DNA chromosome Ib	96%

**Table 4:** ***Toxoplasma gondii *IVIAT- identified genes by immunoscreening of cDNA library using anti-human IgG-HRP**

**GenBank ID/accession no.**		**Gene ID in Toxo DB**	**Gene name**	**Description**	**Homology percentage**
**1**	7901108	TGME49_069250	26S proteasome regulatory subunit	*Toxoplasma gondii ME49* 26S proteasome regulatory subunit, putative	95%
**2**	7901478	TGME49_097940	hypothetical protein	*Toxoplasma gondiiME49* hypothetical protein	99%
**3**	7894322	TGME49_030160	hypothetical protein	*Toxoplasma gondiiME49* hypothetical protein	93%
**4**	7894761	TGME49_061750	hypothetical protein	*Toxoplasma gondiiME49* hypothetical protein	98%
**5**	7897485	TGME49_005180	U1 small nuclear ribonucleoprotein	*Toxoplasma gondii ME49* U1 small nuclear ribonucleoprotein, putative	95%
**6**	7899395	TGME49_037230	hypothetical protein	*Toxoplasma gondiiME49* hypothetical protein	80%
**7**	7895435	TGME49_024460	aminopeptidase N	*Toxoplasma gondii ME49* aminopeptidase N, putative	82%
**8**	7895096	TGME49_027910	hypothetical protein	*Toxoplasma gondiiME49* hypothetical protein	95%
**9**	7895077	TGME49_025560	hypothetical protein	*Toxoplasma gondiiME49* hypothetical protein	99%
**10**	7897605	TGME49_025080	40S ribosomal protein S18	*Toxoplasma gondii ME49* 40S ribosomal protein S18, putative	84%
**11**	7899217	TGME49_068830	hypothetical protein	*Toxoplasma gondiiME49* hypothetical protein	94%
**12**	AM055942.3	TGME49_095420	hypothetical protein	*Toxoplasma gondii RH* genomic DNA chromosome Ia	87%
**13**	7894058	TGME49_002840	zinc finger (C3HC4 type)	*Toxoplasma gondii ME49* zinc finger (C3HC4 type)/FHA domain-containing protein	97%
**14**	7893896	TGME49_075310	hypothetical protein	*Toxoplasma gondiiME49* hypothetical protein	94%
**15**	7899266	TGME49_047530	hypothetical protein	*Toxoplasma gondiiME49* hypothetical protein	96%
**16**	7893521	TGME49_018520	protein MIC6	*Toxoplasma gondii* micronemal protein MIC6 (MIC6) mRNA, complete	95%
**17**	7893531	TGME49_018720	protein kinase	*Toxoplasma gondii ME49* calcium-dependent protein kinase, putative	91%
**18**	7896164	TGME49_025060	nucleoredoxin	*Toxoplasma gondii ME49* nucleoredoxin, putativ	98%
**19**	7896097	TGME49_026520	hypothetical protein	*Toxoplasma gondiiME49* hypothetical protein	98%
**20**	7901507	TGME49_078510	Protein phosphatase 2C	*Toxoplasma gondii ME49* protein phosphatase 2C, putative	95%
**21**	7894761	TGME49_061750	hypothetical protein	*Toxoplasma gondiiME49* hypothetical protein	98%
**22**	7900407	TGME49_078450	hypothetical protein	*Toxoplasma gondiiME49* hypothetical protein	95%
**23**	7897848	TGME49_095620	zinc finger (CCCH type)	*Toxoplasma gondii ME49* zinc finger (CCCH type) protein, putative	91%
**24**	7900113	TGME49_035990	hypothetical protein	*Toxoplasma gondiiME49* hypothetical protein	91%
**25**	7899268	TGME49_045880	hypothetical protein	*Toxoplasma gondiiME49* hypothetical protein	88%
**26**	7893794	TGME49_067740	hypothetical protein	*Toxoplasma gondiiME49* hypothetical protein	99%
**27**	7893464	TGME49_071050	surface antigen P22	*Toxoplasma gondii strain 14* surface antigen P22 3′ flanking sequence	85%
**28**	7899477	TGME49_049670	cysteine proteinase	*Toxoplasma gondii ME49* cysteine proteinase (cp1) mRNA, complete cds	95%
**29**	AY424892.1	AY424892.1	UDP-N-acetyl-D-galactosamine	*Toxoplasma gondii* UDP-N-acetyl-D galactosamine:polypeptide N-acetylgalactosaminyltransferase T3 gene, complete cds	90%

### Assessment of IVIAT-identified gene expression by real-time PCR

**Figure 2:**
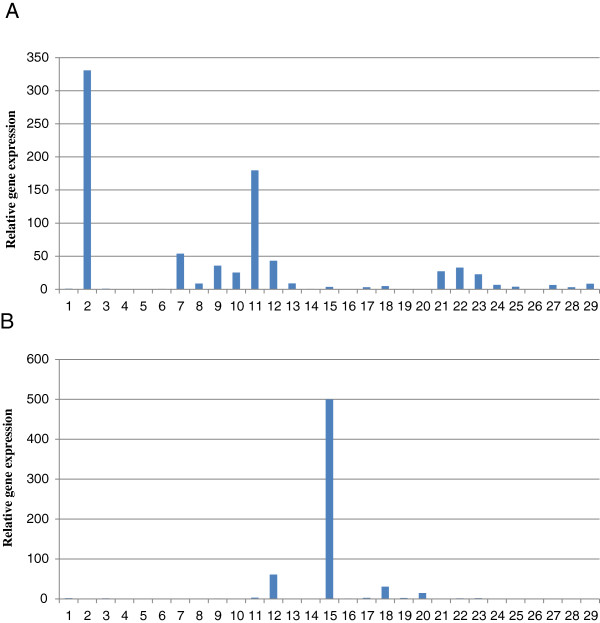
**Relative gene expression level (*in-vivo *to *in-vitro*) for *T.gondii *genes identified by IVIAT. (A).** Genes detected by IgM antibody **(B).** Genes detected by IgG antibody. The y axis presented the identified genes by number.

Due to the large number of samples in this study and the use of different control samples for each round of experiments, the detailed data could not be placed in one table. Therefore only the fold changes of the examined sequences are presented in Tables 5 and 6 to show the relative expression levels between *in-vivo* and *in-vitro* grown parasites for IgM-detected sequences and IgG-detected sequences respectively. Of the 29 IgM-detected genes analyzed, 20 (69%) were up-regulated *in-vivo* relative to their *in-vitro* expression levels with fold changes between 3.11 and 330.8 (Figure 2A). Table 5 shows the fold changes of the expression of the genes; the results indicated that there were significant increases, ranging from 3.11 to 330.8-folds of the gene expression levels of *in-vivo* over *in-vitro* grown parasites for 20 of 28 IgM-detected genes. Of the 29 IgG-detected genes analyzed, 10 (34%) were up-regulated *in-vivo* relative to their expression levels *in-vitro* with fold changes between 1.59 and 499.86 (Figure 2B). Table 6 shows the fold changes of the expression of the genes; the results indicated that there were significant increases, ranging from 1.31 to 449.86-folds of the expression levels of *in-vivo* over *in-vitro* for 11 of 29 IgG-detected genes.

**Table 5:** **Relative mRNA expression levels of *T.gondii *IVIAT-identified genes detected by IgM using 2**^**-**^ΔΔ^**Ct**^**method**

**No**	**GenBank ID**	**Fold change**	**Protein encoded by gene**
**1**	7895406	0.86	
**2**	7898829	330.8	hypothetical protein
**3**	7894091	0.97	
**4**	7900912	0.14	
**5**	7899945	0.00048	
**6**	7893976	0.6	
**7**	7893824	53.81	sterol-regulatory element binding protein site
**8**	7899621	8.6	
**9**	7901413	35.75	microneme
**10**	7894150	25.28	hypothetical protein
**11**	7895338	179.76	hypothetical protein
**12**	7898197	43.11	hypothetical protein
**13**	7895974	8.87	
**14**	7900011	0.62	
**15**	L06091.1	3.6	
**16**	7896316	0.3	
**17**	7894239	3.22	
**18**	7896580	4.89	
**19**	7899034	0.09	
**20**	7899034	0.26	
**21**	AY707938.1	27.28	SRS34A
**22**	7894324	32.89	hypothetical protein
**23**	AF364813.1	22.6	MIC2-associated protein M2AP
**24**	7900910	6.82	
**25**	7895521	3.78	
**26**	7896316	0.24	
**27**	7897539	6.49	
**28**	7895127	3.11	
**29**	7896796	8.5	

**Table 6:** **Relative mRNA expression levels of *T.gondii *IVIAT-identified genes detected by IgG using 2**^**-**^ΔΔ^**Ct **^**method**

**No**	**GenBank ID**	**Fold change**	**protein encoded by gene which are upregulated >10 ×**
**1**	7901108	1.67	
**2**	7901478	0.80	
**3**	7894322	1.31	
**4**	7894761	0.33	
**5**	7897485	0.36	
**6**	7899395	0.05	
**7**	7895435	0.01	
**8**	7895096	0.16	
**9**	7895077	0.98	
**10**	7897605	0.18	
**11**	7899217	3.03	
**12**	AM055942.3	60.97	hypothetical protein
**13**	7894058	0.01	
**14**	7893896	0.05	
**15**	7899266	499.86	hypothetical protein
**16**	7893521	1	
**17**	7893531	2.84	
**18**	7896164	30.70	nucleoredoxin
**19**	7896097	2.15	
**20**	7901507	14.86	Protein phosphatase 2C
**21**	7894761	0.18	
**22**	7900407	1.59	
**23**	7897848	1.75	
**24**	7900113	0.1	
**25**	7899268	0.08	
**26**	7893794	0.23	
**27**	7893464	0.01	
**28**	7899477	0.02	
**29**	AY424892.1	0.02	

### Sensitivity and specificity evaluation

**Figure 3:**
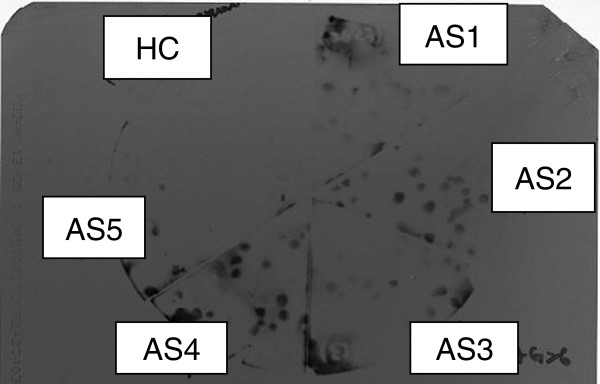
**Representative results of sensitivity evaluation of clone which expressed sterol-regulatory element binding protein site.** AS1-AS4: serum from persons with acute toxoplasmosis. HC: serum from healthy person.

The top three most up-regulated IgM- and IgG-detected clones were subjected to sensitivity and specificity analysis. Figure 3 shows an example of the immunoblot results, and Table 7 shows the summary of the results. High sensitivity (90.0%) was observed for three IgM-detected clones and one of the IgG-detected clones. However the specificity was generally not high, the best was a moderate specificity of 70% from a hypothetical protein expressed by an IgM-detected clone (GenBank accession no. 7898829).

**Table 7:** Sensitivity and specificity results for three up-regulated IgM and IgG-detected clones identified by IVIAT

**GenBank ID**	**Fold change**	**Sensitivity**	**Specificity**	**Antibody detection**
**7898829**	330.8	90% (9/10)	70% (7/10)	IgM
**7893824**	53.81	90% (9/10)	50% (5/10)	IgM
**7895338**	179.76	90% (9/10)	40% (4/10)	IgM
**AM055942.3**	60.97	60% (6/10)	60% (6/10)	IgG
**7899266**	499.86	60% (6/10)	70% (7/10)	IgG
**7896164**	30.70	90% (9/10)	60% (6/10)	IgG

## Discussion

IVIAT has been applied in the study of several pathogenic microorganisms based on the assumption that conventional genetic and biochemical *in-vitro* approaches cannot mimic the real complex and dynamic *in-vivo* situation of human infection [19-28]. In our recent published report, *T.gondii* genes preferentially expressed *in-vivo* were identified via immunoscreening of *T.gondii* expression library with sera from chronically-infected individuals followed by real time PCR to confirm the up-regulation of the identified genes. There were increased expression levels of RNA of three genes i.e. SRS3 and two hypothetical protein genes from *in-vivo* grown *T.gondii* relative to the same genes from *in-vitro* grown parasite [13]. This approach does not reflect all of the antigens that are produced *in-vivo* by the eukaryotic parasite, considering that a prokaryotic expression system was used in constructing the cDNA library. Nevertheless, this approach would be expected to identify some of the *in-vivo* induced antigens of the parasite [13].

The rationale for use of *in-vitro* instead of *in-vivo* grown *T. gondii* has been explained in our previous report [13]. A large quantity of ‘clean tachyzoites’ was needed to make a good high titre *T. gondii* cDNA library. As compared to the use of mice as host, the *in-vitro* method had less contamination and produced higher quantity of parasites in a shorter period of time. Although all the RNA may not be exactly the same between *in-vivo* and *in vitro* grown parasites, there should be substantial similarities between them to enable us to observe difference in expression levels between them. Moreover IVIAT was introduced in order to reduce the need to use animals. There are also many other IVIAT studies which used *in-vitro* grown organisms [16,20,29,30]. For the same reasons (i.e. better purity and yield), we used *T. gondii* RH strain instead of ME49 strain, although the available genome information was derived from the latter.

The main serological marker for the diagnosis of acute toxoplasmosis is specific IgM antibody, along with IgG antibody of low avidity [31]. In the present study 29 *T. gondii* sequences were detected by each IgM and IgG secondary antibodies using sera from individuals with serological evidence of acute infection. Due to resource constraints, we did not endeavour to identify all of the IVIAT-detected clones using a high-throughput methodology. However the random selection of the clones for expression analysis by real-time PCR hopefully will provide a ‘snapshot’ of the *in-vivo* induced antigens of *T. gondii.* The results of the transcriptional profiling suggested the induction of genes that have been described as involved in parasite invasion and motility, cellular metabolism, as well as other unknown functions. It is notable that the percentage of up-regulated genes among the IgM-identified clones were twice the percentage of up-regulated genes among the IgG-identified clones. It is also interesting to note that among the IgM and IgG-detected proteins, most were hypothetical proteins namely 14 of 29 and 16 of 29 respectively. Limited sensitivity and specificity evaluation of the top three most up-regulated IgM- and IgG-detected clones showed that none had good specificity, thus further testing with more serum samples were not performed.

Three different microneme proteins namely MIC6, MIC11, MIC13 and a MIC2 associated protein were identified in this study. MIC6 showed no difference in the mRNA expression levels *in-vivo* over *in-vitro,* but the other three genes detected by IgM (MIC11, MIC13 and MIC2 associated protein) showed significant increases of mRNA expression level of *in-vivo* grown *T.gondii* in comparison to *in-vitro* grown *T. gondii*. Micronemes are sub-cellular organelles that belong to apicomplexan protozoans which secrete proteins (MICs) known to be responsible for the gliding motility and subsequent invasion process of *T. gondii*[32]. MIC11 is a conserved microneme protein in apicomplexan protozoans that is secreted in a calcium dependent manner while MIC13 has been reported as a successful vaccine candidate against chronic infection and congenital toxoplasmosis in mice [33,34]. In this study, the increased expression levels of MIC11, MIC13 and a MIC2 associated protein mRNA observed in the *in-vivo* grown tachyzoites may be due to their role in attachment and invasion of the tachyzoites in the host during acute infection.

Meanwhile, sequences of DNA chromosome Ιb were detected by IgM whereby three sequences which code a dynamic-like protein, a hypothetical protein and cyclin-N terminal domain showed increased level of mRNA expression of *in-vivo* grown over *in-vitro* grown *T. gondii*. Besides, a high increase in the mRNA expression levels (60.67 fold change) was observed for DNA chromosome Ιa sequence that was detected by IgG. Comparative studies between the chromosome genomes of *T. gondii* RH Type I and ME49 Type II strains revealed polymorphisms at a frequency of ~1 in 100 bp among all chromosomes except chromosome Ia (chrIa). Chromosome Ia has the same gene content and structural elements in both strains and this suggest that chrIa has monomorphic inheritance compared to the rest of the *T. gondii* chromosomes for all three clonal lineages although the biological basis remains unknown. The unusual monomorphism of chrIa has led us to the conclusion that genes located on this chromosome may allow some fitness advantage [35].

Eleven other genes with more than 10 times fold changes include nucleoredoxin, protein phosphatase 2C, sterol regulatory protein, surface antigen P22 3’ flanking sequence [SRS34A (= SAG2A, P22)] and six ORF coding hypothetical proteins. We were most intrigued by the observation of a very high expression level for one of the up-regulated genes detected by IgG, with *in-vivo* over *in-vitro* fold change of 499.86. Unfortunately the function of the gene is unknown. Nucleoredoxin (NRX) is a novel gene which encodes a protein similar to ATrx1, a member of thioredoxins (TRX) family. Nucleoredoxin is highly conserved in all the kingdoms of living organisms and was formerly reported to be nuclear and more recently cytosolic. It appears to play a role in signal transduction and may be a redox regulator factor of the nuclear proteins [36,37].

Protein phosphatase 2C (PP2C) is a Mn2+ or Mg2 -dependent protein Ser/Thr phosphatase secreted by rhoptry, a secretory organelle of *T. gondii*. It was identified in a proteomic survey of rhoptry proteins (ROPs), and found to be essential for the invasion process of apicomplexan parasites [38]. Host cell invasion and motility in apicomplexa protozoa occurs through actin polymerization as the main process. Actin monomers and their cap filaments in *T. gondii* are secreted by toxofilins and its activities *in-vitro* and *in-vivo* are dependent on phosphorylation of this protein. Recently a type 2C phosphatase has been reported to be involved in toxifilin phosphorylation and activity [39].

Sterol regulatory element-binding proteins (SREBPs) were found to be one of the three most up-regulated sequences detected by IgM in this study. SREBPs are basic-helix-loop-helix leucine zipper class of transcription factors which are specific for fatty acid and cholesterol synthesis. When the level of sterols is low, SREBPs are cleaved to a water soluble N-terminal domain which is then translocated to the nucleus [40].

SRS34A (= SAG2A, P22), is a plasma membrane gene of *T. gondii* found during and after tachyzoite invasion. SAG family of proteins which cover the surface of *T. gondii* bradyzoites and tachyzoites comprise five main members i.e. SAG1 (P30 kDa), SAG2 (P22 kDa), SAG3 (P43 kDa), SAG4 (P23 kDa) and SAG5 (P35 kDa) [41,42]. SAG1 and SAG2A are immunodominant within the superfamily and induce a high antibody response in early infection. The increased mRNA expression level of SRS34A (SAG2A) which has been detected by IgM in this study is in agreement with other studies which reported that tachyzoite surface is dominated by SAG1, SAG2A, SAG3, SRS1, SRS2 and SRS3 and have been shown to be involved in attachment and invasion necessary for early acute *T. gondii* infection [43].

## Conclusion

This study identified several *T. gondii* genes with up-regulated expression *in-vivo* as compared to *in-vitro*. Further studies including stage-specific expression and localisation studies on the highly up-regulated genes and the corresponding proteins, in particular the hypothetical proteins, should be performed. This may help to increase our understanding of the disease pathogenesis and asses their usefulness as potential vaccine candidates.

## Competing interests

The authors have no conflict of interests in this article.

## Authors’ contributions

All authors participated in writing and editing of the manuscript. RN: designed and supervised the whole study, supervised the data analysis and interpretation and the manuscript writing, performed the major editing of the manuscript, and acquired funding for the project. AA: performed the experiments and data analysis, which encompassed serum pre-absorption, cDNA library immunoscreening, *in-vivo* excision, plasmid purification and real-time PCR expression analysis. Also wrote the first draft of the manuscript. BYK: supervised the real-time PCR and the interpretation of the results. AYT: performed the sensitivity and specificity studies of the selected clones. MG: provided the acute toxoplasmosis serum samples, performed the initial serological analysis of the samples, participated in the data analysis and interpretation. IZAK and MHY: maintained the animals and cell-culture for growing *T. gondii in-vivo* and *in-vitro* respectively; and collected the *in-vitro* and *in-vivo T. gondii*. SO: performed the serological anlaysis of the serum samples (IgM, IgG, IgG avidity). All authors read and approved the final manuscript.

## Pre-publication history

The pre-publication history for this paper can be accessed here:

http://www.biomedcentral.com/1471-2334/13/287/prepub
